# Transforming Virtual Healthcare: The Potentials of ChatGPT-4omni in Telemedicine

**DOI:** 10.7759/cureus.61377

**Published:** 2024-05-30

**Authors:** Mohamad-Hani Temsah, Amr Jamal, Khalid Alhasan, Fadi Aljamaan, Ibraheem Altamimi, Khalid H Malki, Abdulrahman Temsah, Robin Ohannessian, Ayman Al-Eyadhy

**Affiliations:** 1 Pediatric Intensive Care Unit, Pediatric Department, King Saud University Medical City, College of Medicine, King Saud University, Riyadh, SAU; 2 Family and Community Medicine, King Saud University, Riyadh, SAU; 3 Pediatric Nephrology, King Saud University, Riyadh, SAU; 4 Critical Care Department, College of Medicine, King Saud University, Riyadh, SAU; 5 College of Medicine, King Saud University, Riyadh, SAU; 6 Department of Otolaryngology, College of Medicine, King Saud University, Riyadh, SAU; 7 Software Engineering Department, College of Engineering, Alfaisal University, Riyadh, SAU; 8 Digital Health, Vickino Institute of Telehealth, Vickino, ARM; 9 Department of Pediatrics, Pediatric Intensive Care Unit, College of Medicine, King Saud University, Riyadh, SAU; 10 Pediatric Intensive Care Unit, King Saud University Medical City, Riyadh, SAU

**Keywords:** patient communication, digital health innovations, ai in healthcare, large language models (llm), health information privacy, data privacy, natural language processing, virtual healthcare, telemedicine, chatgpt-4o

## Abstract

The introduction of OpenAI's ChatGPT-4omni (GPT-4o) represents a potential advancement in virtual healthcare and telemedicine. GPT-4o excels in processing audio, visual, and textual data in real time, offering possible enhancements in understanding natural language in both English and non-English contexts. Furthermore, the new "Temporary Chat" feature may improve privacy and data confidentiality during interactions, potentially increasing integration with healthcare systems. These innovations promise to enhance communication clarity, facilitate the integration of medical images, and increase data privacy in online consultations. This editorial explores some future implications of these advancements for telemedicine, highlighting the necessity for further research on reliability and the integration of advanced language models with human expertise.

## Editorial

**Figure 1:**
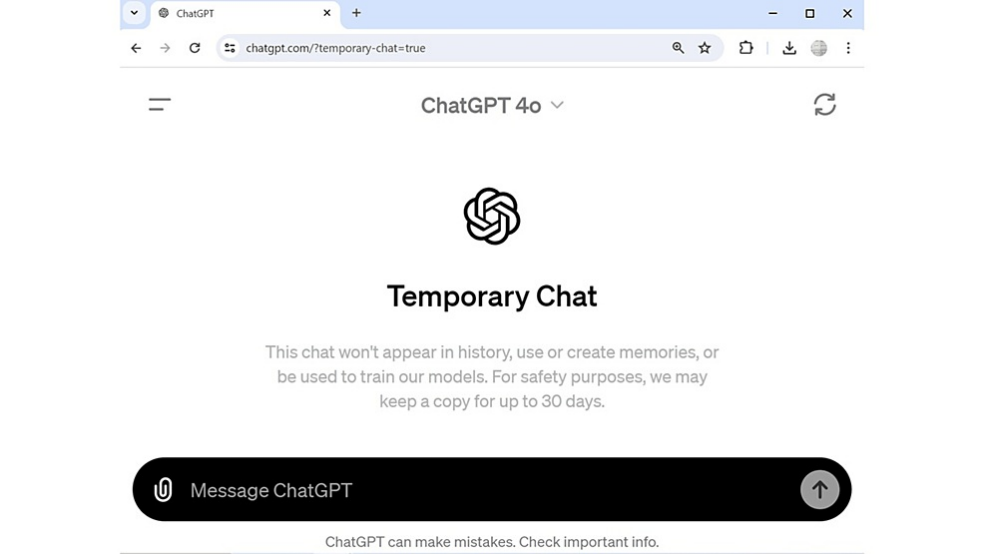
Screenshot taken on May 16, 2024, from the desktop version of ChatGPT-4o in the “Temporary Chat” model.

On May 13, 2024, OpenAI announced their latest ChatGPT model, GPT-4o (“o” for “omni”), as their latest flagship model that could interact with users and analyze multiple audios, vision, and text, all in real-time fashion [1]. In the ever-evolving landscape of digital health, the introduction of ChatGPT-4o could mark another advancement, with the potential to enhance telemedicine's effectiveness and accessibility [2,3]. OpenAI's latest iteration of its large language model (LLM) brings two groundbreaking features: the GPT-4o and advanced natural language understanding and processing. Furthermore, the addition of the valuable feature of “Temporary Chat” which was also introduced in February 2024, allows additional privacy in the chat functionality (Figure 1). These innovations may have additional attractive capabilities to incorporate into telemedicine, reform patients’ remote care, and modify the format of medical consultation format.

The widely spreading and improved natural language processing capabilities of ChatGPT-4o are promising to enable richer interactive, faster response, and seamless multilingual understanding between human users and LLM when handling medical queries [1]. This may enable patients to receive clearer, more precise responses, which is crucial in a field where clarity can significantly impact health outcomes. Integrating such improved LLM in telemedicine would improve the experience, especially keeping in mind that cases discussed through telemedicine are usually complicated ones. With major advancements in handling text in both English and non-English languages, GPT-4o is also much faster, allowing better bidirectional communication, that is well-suited for virtual online consultations, that may incorporate the patient, healthcare provider, and GPT-4o in the same live discussions. Additionally, as the GPT-4o excels in vision and audio understanding compared to previous models, this online consultation could provide a vivid experience for both the patient and the healthcare provider.

Users could upload medical images or files, so the LLM could analyze them and incorporate them into its decision output. While previous ChatGPT-4 could interact with audio and visual input before, the new feature of using a mobile phone's camera in “live” interaction enriches interactivity for users [4]. The ChatGPT-4o's ability to comprehend and respond to complex medical terminology and patient concerns with higher accuracy may reduce the risk of miscommunication and enhance the reliability of online consultations. More research on capabilities is necessary as patients and healthcare providers start using these advanced technologies, keeping in mind the possibility of errors or “hallucinations” that may be integrated into parts of these human-ChatGPT-4o interactions, as the model itself states that "ChatGPT can make mistakes" (Figure 1) [5,6]. Therefore, expert human medical oversight is essential rather than optional. The ability to produce a timely consultation summary that should be approved by the consultant is another enabler for effective communication and documentation for the telemedicine session.

The “Temporary Chat” feature is another feature added by OpenAI that was launched in mid-February 2024 that may allow users to interact with the ChatGPT models more privately during medical consultations [7]. This feature ensures that conversation content is not used to train the models or stored in the user chat history, addressing significant privacy, medicolegal, and ethical concerns that were raised previously [8-10]. However, as Figure 1 illustrates, OpenAI retains the data for up to 30 days for operational purposes, raising important considerations for data confidentiality and transparency regarding storage and access. While this “invisible” chat can provide real-time assistance, suggest diagnoses and potential treatment plans, and answer medical questions, the data privacy feature is still not clear at this stage; therefore, it warrants continued vigilance when sharing patient and healthcare data in this setting until Health Insurance Portability and Accountability Act (HIPAA)-compliant LLM protocols are certified to guard data privacy [11]. On the other hand, it would also be relevant for a medical AI platform to keep previous interactions with a patient to provide more personalized answers.

From a telemedicine perspective, these ChatGPT-4o and the “Temporary Chat” capabilities may boost efficiency and patient satisfaction [12]. Patients can have their concerns addressed promptly and possibly more accurately in their native language [13]. Physicians and other healthcare providers, on the other hand, can manage their time more effectively, attending to a larger number of patients with the support of an AI assistant that supports their expertise. Furthermore, advanced LLMs could enhance personalized healthcare provision, which may be supplemented with updated global healthcare studies, such as the Global Burden of Disease, giving healthcare professionals another tool for making more tailored medical decisions using rapidly evolving data [14]. The cost of accessing healthcare expertise through telemedicine platforms may also be impacted by the ubiquitous accessibility of such knowledge.

Furthermore, the scalability of AI solutions like ChatGPT-4o means that high-quality telemedicine services can reach underserved and remote areas, bridging the gap in healthcare access [1,12]. As LLMs continue to learn and rapidly evolve, their application in telemedicine could optimize care, ensuring that all patients receive high-quality consultations regardless of their location. We hereby propose a new three-pillar online consultation integration, with the patient, healthcare professional, and GPT-4o sharing and discussing their insights in real-time conversation. Despite the significant potential, physicians must oversee LLM development and public perceptions, advocating for its proper use to ensure patient care is not compromised [15]. Supporting multiple human languages could also be a valuable tool for such remote consultations, where insightful healthcare advice could be provided clearly to patients in their native language, making it more widespread and fulfilling participants’ needs without language barriers [16].

However, it is essential to acknowledge the limitations of this evolving technology. Despite its advanced capabilities, research into the healthcare-related reliability of ChatGPT-4o warrants further research. Previous ChatGPT and generative text-to-images models, like DALL· E 3, demonstrated instances of incorrect or incomplete responses, particularly in highly specialized, complicated, or ambiguous medical cases [5,17]. Moreover, the AI's dependence on pre-existing data means it might not always incorporate the most updated medical research or emerging treatments. Therefore, while ChatGPT-4o can be a powerful tool, it should complement rather than replace human expertise, with healthcare professionals making the final decisions in patient care. At the same time, it is important to stress that it would be difficult to research all medical situations to validate these models with clinical studies and that healthcare professionals should always remain aware of the potential limitations.

Another point to address is the impact of such technology on costs in both directions. On one hand, it can decrease costs due to improved time efficiency; on the other hand, it might increase costs due to unnecessary investigations prompted by AI-suggested diagnoses. Studies have shown that while telehealth can streamline certain processes, its implementation often requires substantial upfront investment, which may not immediately translate to cost savings [18]. Furthermore, the potential anxiety that may be provoked by AI-generated suggestions could consume more time and medical investigations as healthcare professionals work to reassure patients in these scenarios. Therefore, there is a continuous need for monitoring and validating widespread LLM recommendations to avoid unnecessary tests and treatments, making ongoing surveillance and stakeholders’ involvement a crucial part of managing overall telemedicine expenses.

## References

[1] (2024). Open AI: How can I access GPT-4, GPT-4 Turbo and GPT-4o?. https://help.openai.com/en/articles/7102672-how-can-i-access-gpt-4-gpt-4-turbo-and-gpt-4o..

[2] Liu J, Wang C, Liu S (2023). Utility of ChatGPT in clinical practice. J Med Internet Res.

[3] Ohannessian R, Fortune N, Moulin T, Madden R (2020). Telemedicine, telestroke, and artificial intelligence can be coded with the International Classification of Health Interventions. Telemed J E Health.

[4] Temsah R, Altamimi I, Alhasan K, Temsah MH, Jamal A (2023). Healthcare's new horizon with ChatGPT's voice and vision capabilities: a leap beyond text. Cureus.

[5] Alkaissi H, McFarlane SI (2023). Artificial hallucinations in ChatGPT: implications in scientific writing. Cureus.

[6] Athaluri SA, Manthena SV, Kesapragada VS, Yarlagadda V, Dave T, Duddumpudi RT (2023). Exploring the boundaries of reality: investigating the phenomenon of artificial intelligence hallucination in scientific writing through ChatGPT references. Cureus.

[7] (2024). Open AI: Memory and new controls for ChatGPT. https://openai.com/index/memory-and-new-controls-for-chatgpt/..

[8] Ong JCL, Chang SY, William W (2024). Ethical and regulatory challenges of large language models in medicine. Lancet Digit Health.

[9] Karabacak M, Margetis K (2023). Embracing large language models for medical applications: opportunities and challenges. Cureus.

[10] Temsah MH, Aljamaan F, Malki KH (2023). ChatGPT and the future of digital health: a study on healthcare workers' perceptions and expectations. Healthcare (Basel).

[11] Sheth S, Baker HP, Prescher H, Strelzow JA (2024). Ethical considerations of artificial intelligence in health care: examining the role of generative pretrained transformer-4. J Am Acad Orthop Surg.

[12] Snoswell CL, Snoswell AJ, Kelly JT, Caffery LJ, Smith AC (2023). Artificial intelligence: Augmenting telehealth with large language models. J Telemed Telecare.

[13] Teixeira da Silva JA (2023). Can ChatGPT rescue or assist with language barriers in healthcare communication?. Patient Educ Couns.

[14] Temsah MH, Jamal A, Aljamaan F, Al-Tawfiq JA, Al-Eyadhy A (2023). ChatGPT-4 and the global burden of disease study: advancing personalized healthcare through artificial intelligence in clinical and translational medicine. Cureus.

[15] Beltrami EJ, Grant-Kels JM (2024). Consulting ChatGPT: Ethical dilemmas in language model artificial intelligence. J Am Acad Dermatol.

[16] Nguyen MT, Garcia F, Juarez J (2022). Satisfaction can co-exist with hesitation: qualitative analysis of acceptability of telemedicine among multi-lingual patients in a safety-net healthcare system during the COVID-19 pandemic. BMC Health Serv Res.

[17] Temsah MH, Alhuzaimi AN, Almansour M (2024). Art or artifact: evaluating the accuracy, appeal, and educational value of AI-generated imagery in DALL·E 3 for illustrating congenital heart diseases. J Med Syst.

[18] Tukur M, Saad G, AlShagathrh FM, Househ M, Agus M (2023). Telehealth interventions during COVID-19 pandemic: a scoping review of applications, challenges, privacy and security issues. BMJ Health Care Inform.

